# Niche Overlap of Congeneric Invaders Supports a Single-Species Hypothesis and Provides Insight into Future Invasion Risk: Implications for Global Management of the *Bactrocera dorsalis* Complex

**DOI:** 10.1371/journal.pone.0090121

**Published:** 2014-02-27

**Authors:** Matthew P. Hill, John S. Terblanche

**Affiliations:** 1 Conservation Ecology & Entomology Department, Faculty of AgriSciences, Stellenbosch University, Western Cape, South Africa; 2 Centre for Invasion Biology, Conservation Ecology & Entomology Department, Faculty of AgriSciences, Stellenbosch University, Western Cape, South Africa; Instituto de Higiene e Medicina Tropical, Portugal

## Abstract

**Background:**

The invasive fruit fly, *Bactrocera invadens*, has expanded its range rapidly over the past 10 years. Here we aimed to determine if the recent range expansion of *Bactrocera invadens* into southern Africa can be better understood through niche exploration tools, ecological niche models (ENMs), and through incorporating information about *Bactrocera dorsalis* s.s., a putative conspecific species from Asia. We test for niche overlap of environmental variables between *Bactrocera invadens* and *Bactrocera dorsalis* s.s. as well as two other putative conspecific species, *Bactrocera philippinensis and B. papayae.* We examine overlap and similarity in the geographical expression of each species’ realised niche through reciprocal distribution models between Africa and Asia. We explore different geographical backgrounds, environmental variables and model complexity with multiple and single *Bactrocera* species hypotheses in an attempt to predict the recent range expansion of *B. invadens* into northern parts of South Africa.

**Principal Findings:**

*Bactrocera invadens* has a high degree of niche overlap with *B. dorsalis* s.s. (and *B. philippinensis* and *B. papayae*). Ecological niche models built for *Bactrocera dorsalis* s.s. have high transferability to describe the range of *B. invadens*, and *B. invadens* is able to project to the core range of *B. dorsalis* s.s. The ENMs of both *Bactrocera dorsalis* and *B. dorsalis* combined with *B. philipenesis* and *B. papayae* have significantly higher predictive ability to capture the distribution points in South Africa than for *B. invadens* alone.

**Conclusions/Significance:**

Consistent with other studies proposing these *Bactrocera* species as conspecific, niche similarity and overlap between these species is high. Considering these other *Bactrocera dorsalis* complex species simultaneously better describes the range expansion and invasion potential of *B. invadens* in South Africa. We suggest that these species should be considered the same–at least functionally–and global quarantine and management strategies applied equally to these *Bactrocera* species.

## Introduction

Alien invasive invertebrate species represent some of the most recognized vectors of agricultural damage [1], as well as important vectors of disease [2], [3]. Invasions of such pests are increasingly driven by anthropogenic movements, particularly trade. After overcoming a geographic or dispersal invasion barrier, typically facilitated by high levels of propagule pressure (reviewed in [4], [5]), and presuming non-limiting biotic interactions (e.g. host availability and lack of competition), the establishment success and subsequent distribution and abundance of an invasive species is ultimately determined by the species relationship to abiotic variables such as climate (e.g. [6], [7]). These relationships can be interpreted through the concept of the niche [8] and has led to the advent of species distribution models in the form of ecological niche models (ENMs) to predict the establishment and spread of invasive species [9], [10]. Typically, ENMs approximate something close to the realised niche of the species [11] through characterizing species-environment relationships across a known distribution [12]. The models can then be extrapolated or projected to new geographic space (e.g. [13]–[15]) to investigate potential of invasion [16], and may provide information to promote risk status and aid management decisions (e.g. [17], [18]). In addition to predicting invasion potential, ENMs can also be used as exploratory tools to examine niche similarity and divergence between taxonomically uncertain species (e.g. [19]). Ecological niche models have been used to help identify niche boundaries of congeneric and cryptic species (e.g. [20], [21]), and in a similar way it should be possible to use ENMs to test taxonomic boundaries of invasive species (e.g. [22]–[23]), leading to recommendations for pest control or management within global trade and tourism networks.

A major challenge for applying ENMs to alien invasive species is that environmental limits may be different in native and invasive ranges resulting in asymmetrical transference of models [24]. For instance, when characterizing the realised niche of the native range, the species may be inhibited by a range of barriers, including biotic and abiotic factors, that do not exist in the invasive range [25] resulting in underestimation of the potential invasive niche. Further, alien invasive species are often not in a state of equilibrium with their environment, particularly within the novel, invaded range [16], [26]. This may translate into geographic range expansions as species continue to spread to fill their potential niche [6], [7], or are enabled to do so through niche shifts (e.g. [27]), which may be facilitated by evolutionary adaptation (e.g. [28]). In the absence of strong biotic interactions however, it is possible to explore modelled responses and apply ENMs in an attempt to account for unstable relationships with climate, and as yet unencountered environmental conditions (e.g. [26]). To help accurately predict the extent of an invasion using ENMs, species-environment relationships in both the native and invasive ranges may need to be characterised [11], [25], [29]. In consequence, characterising the realised niche across both native and invasive ranges first requires that taxonomic and functional species boundaries are effectively described. For example, species descriptions may differ between countries or continents, especially in the case of cryptic species or life-stages, so that only distribution points corresponding to a particular description are employed in modelling attempts: such sub-taxon level modelling is likely to result in predictions different from ENMs considering a broader realised niche [22], [25], [30].

Fruit flies (Diptera: Tephritidae) are major economic pests through the world, causing huge economic losses to production of a wide range of commercial fruits. Some of the most economically important members of this family are within the *Bactrocera dorsalis* complex, comprising ∼75 species. Two members of this complex, *Bactrocera dorsalis* s.s. and *Bactrocera invadens*, are highly polyphagous pests of a variety of plant species, with 250 identified hosts for *B. dorsalis* s.s. [31] and over 43 for *B. invadens* [32]. *Bactrocera dorsalis* s.s. is thought to have originated in northern southeast Asia and has since expanded its range through subtropical Asia and the Pacific Ocean [31], [33]. After detection in East Africa in 2003, *Bactrocera invadens* was described as a separate species from *B. dorsalis* [34] and those invasive populations are thought to have a Sri Lankan origin [32], [34].

Besides subtle morphological characters [34], there is little evidence to functionally separate *B. invadens* from *B. dorsalis.* For example, Khamis *et al.* [32] examined morphometry and DNA barcoding to demonstrate that *B. invadens* is more closely related to *B. dorsalis* than other *Bactrocera* species in that analysis. Further, Tan *et al*. [35] found no difference between phenylpropanoid metabolites (sex pheromones) in *B. invadens* and *B. dorsalis* males, and concluded they are a single species. While other *B. dorsalis* complex members are also considered separate species, recent molecular information has revealed little or no tangible species boundaries between some representatives of this complex (e.g. [32], [33]) and random mating occurs readily between the investigated pairs [36]. Recent studies have examined the invasion potential of both *B. dorsalis* s.s. [37] and *B. invadens* [38] separately, using a fitted-process based model (CLIMEX) and ENMs (Maxent and GARP) respectively. De Meyer *et al.* [36] proposed that “*the climatic optimal conditions for the two species* [*B. dorsalis* and *B. invadens*] *likely overlap broadly*”. Since these modelling attempts, *B. invadens* has undergone rapid range expansion to establish in areas thought to be marginally climatically suitable, and is now reportedly present in the Limpopo province of South Africa [39], after repeated incursions and eradication reported from 2010 [40]. This *B. invadens* range expansion may reflect changes in drivers such as a climatic niche shift or increased propagule pressure, or that *B. dorsalis* and *B. invadens* have been considered separately, as opposed to a single species now fulfilling its potential niche.

These four *Bactrocera dorsalis* complex members provide an opportunity to understand niche differentiation between cryptic or conspecific species, and gain insight into biological invasions and range expansions more generally. Here we address three key questions which we answer through combining different niche exploratory methods and ENMs. First, do *Bactrocera dorsalis* and *Bactrocera invadens* display high niche overlap, and does this provide support for a single-species hypothesis (c.f. [33], [35], [36])? Second, is the recent range expansion of *B. invadens* into southern Africa likely due to niche shift, or is the species simply filling the realised niche which would have been predictable from including information from the range of *B. dorsalis* (and *B. philippinensis* and *B. papayae*)? Third, given potential information gained from addressing the first two questions, can revised ENMs for *Bactrocera* spp. provide better predictions of global invasion potential, and in turn, recommendations for management? Through addressing these questions we therefore aim to better understand niche overlap and species boundaries among *Bactrocera* species, range expansions and biological invasion processes in general, and direct future research to investigate key functional and phenological traits to understand outbreak potential and persistence of these important fruit fly pests.

## Materials and Methods

### Distribution Data

**Figure 1:**
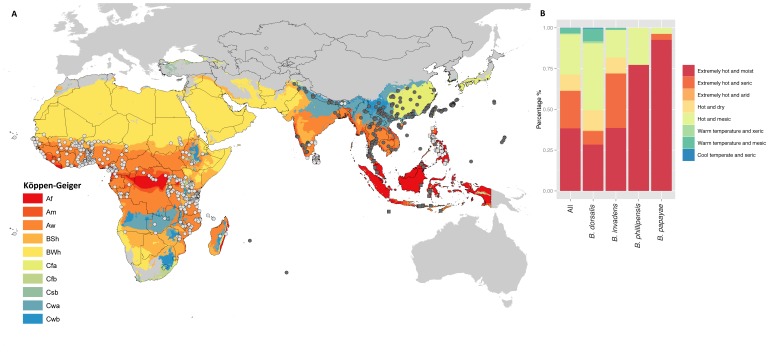
Asian and African distributions of *Bactrocera* spp. a) *Bactrocera dorsalis* s.s (grey circles), *Bactrocera invadens* (white circles), *Bactrocera philippinensis* (grey squares), *Bactrocera papayae* (white squares). Grey area represents area not used for background selection. Colours refer to Köppen-Geiger classifications for presence records of each species investigated. Af = tropical rainforest; Am = tropical monsoon; tropical wet and dry or savannah climate; BSh = arid steppe climate; BWh = arid desert climate; Cfa = humid, subtropical; Cfb = Oceanic, highlands; Cwa = humid, subtropical; Cwb = temperate highland climate. Black outlines represent administrative boundaries selected prior to climate zone selection. b) Species occupation of different GEnS strata classifications (see [52]). All = all four species combined.

Distribution points for *B. dorsalis* s.s., *B. invadens*, *B. papayae* and *B. philippinensis* were collated from published studies [37], [38], [41]–[46] and contributions from various workers (see acknowledgements). For some localities we were required to georeference the site using Google Earth (version 7.1.1.1888; Google Inc., 2013). Due to this we selected an appropriate scale for our predictor layers (see below) and removed duplicate presence points at the grid cell level. Overall, we obtained 438 points for *Bactrocera invadens*, 243 points for *B. dorsalis* s.s., 22 points for *B. papayae* and 27 points for *B. philippinensis* (Fig. 1a). When considered at the grid cell level, this translates to 390 cells occupied for *B. invadens*, 185 for *B. dorsalis* s.s., and 25 and 19 for *B. philippinensis* and *B. papayae* respectively. This expands on the 236 points used by De Meyer *et al*. [38] in their modelling attempts for *B. invadens*. We considered the following hypotheses of species boundaries: (i) *B. invadens* (ii) *B. dorsalis*, (iii) *B. dorsalis* + *B. papaya* + *B. philippinensis* (iv) *B. dorsalis* + *B. invadens* + *B. papaya* + *B. philippinensis*.

### Background Selection

For ENMs that are constructed within a presence-background framework, the issue of accessible area for the species is important [47], [48]. For broadly distributed invasive species (where dispersal measures are largely unknown) it may be best to select backgrounds based on bioclimatic zones representing little inhibition to accessible area beyond broad climate types. Bioclimatic methods of background selection have also been recommended for their simplicity [48] and practicality [49]. We selected two different backgrounds based on broad (*n* = 30 global bioclimatic zones) and narrow (*n* = 125 global climatic strata) bioclimatic classifications, by determining *Bactrocera* spp. occupancy of different zones (using point localities). For the first background we used the Köppen-Geiger climate classifications (Köppen-Geiger classifications, following the rules defined in [50] as applied to the 5′ resolution WorldClim global climatology (www.worldclim.org; Version 1.4, release 3; [51])). The climate zone types that each dataset encompassed were selected based on presence localities. As the Köppen-Geiger classification has 30 broadly classified zones, it provides a relatively broad background for ENM construction.

Our second background was selected across a finer classification system, using different classes of bioclimate types derived through Principal Components Analysis (PCA) and then a clustering routine to classify principal components into homogeneous strata [52]. This global environmental stratification (GEnS) method has high congruence with the Köppen-Geiger method, though it provides finer resolution through a higher number of classifications (strata; *n* = 125) [52]. Finally, we restricted both backgrounds to appropriate geographical extents. For *B. dorsalis* we restricted the climate zones to Asia and for *B. invadens* we allowed the climate zones to fall in either Africa, or Asia, but not South East Asia. Due to the resolution of our climate layers, our backgrounds did not include small islands such as Hawaii, but the presence information for such small island locations was incorporated into the models.

### Predictor Sets

We obtained environmental data from CliMond [50], which provides 35 bioclimatic variables describing means, seasonality and trends for temperature, precipitation, solar radiation and soil moisture. We used a grid cell resolution of 10′, which is roughly 20×20 km at the equator. We compiled two different predictor sets for each of the *Bactrocera* spp. boundary hypotheses. The first of these was an expert-driven predictor set. Previously, seven of the commonly employed bioclimatic variables were included to construct ENMs for *B. invadens* [38]. These variables describe trends and extremes for temperature and rainfall and were chosen on the basis of them likely reflecting limits to tephritid fly distributions. These variables were also included for other tephritid (*Ceratitis* spp.) fly ENMs [53]. This predictor set consisted of: Mean diurnal temperature range (bio2), Temperature seasonality (standard deviation *100) (bio3), Maximum temperature of warmest month (bio5), Minimum temperature of coldest month (bio6), Temperature annual range (bio7), Precipitation of wettest month (bio13), Precipitation of driest month (bio14), and Precipitation seasonality (coefficient of variation) (bio15). We chose these eight predictor variables as our expert predictor set.

Our second predictor set was derived by first conducting exploratory analysis of the niches for each species. We used Ecological Niche Factor Analysis (ENFA) within the adehabitat package [54] in R (version 3.0.0; 2013 [55]). Some studies have used ENFA to characterize the niche of invasive species and predict distributions (e.g. [16], [56]), though elsewhere ENFA has been suggested to determine variables for inclusion in ENMs [11]. All 35 predictor layers were *z*-transformed and ENFA was conducted for each species and a combined dataset of all species, to examine the utilization of the available environmental variables resulting in two uncorrelated axes, marginality and specialization. Marginality refers to the difference or distance between the total range of environmental variables (accessible area) and the range actually occupied by the species (point localities) [57]. Similarly, specialization refers to the variance of the variables. We used ENFA by calculating marginality for each variable and determining a predictor set that may indicate important limits to the distribution of *Bactrocera* spp., at the scale of climatic variables, with marginality indicating how particular the species is compared to the variable across the whole background provided [57]. Analysis was conducted on both the Köppen-Geiger and GEnS defined backgrounds across both Asia and Africa (see Figure 1a) for each species, to examine the utilization of the environmental space across these backgrounds and determine variable importance based on marginality (values range 0–1). The top ranked variables appropriate to each species dataset were then selected (*n* = 8 to be comparable to expert-driven datasets).

### Niche Overlap

We investigated niche overlap and similarity between the four *Bactrocera* spp. in both environmental (E-space) and geographic (G-space) space. We conducted PCA to summarize our predictor sets into uncorrelated axes at each *Bactrocera* spp. location. For the expert predictor set we included all eight variables. For the ENFA-derived sets we took the eight variables that applied to a combined dataset of all distribution points (see Table 1). We added 1000 random points from the Köppen-Geiger backgrounds of *B. dorsalis* s.s. and *B. invadens* respectively, and then plotted the first two components as a biplot, clustering each of the four species with minimum convex hulls to examine overlap within E- space.

**Table 1:** Variables selected for ENFA predictor sets.

	ENFA scores - Köppen | GEnS									
	All		*B. dorsalis* s.s.		*B. invadens*		*B. papayae*		*B. phillipensis*	
Specialization	0.44	0.40	0.82	0.80	0.59	0.56	2.54	1.60	4.07	3.43
Marginality	1.98	1.69	2.47	2.24	1.84	1.55	3.42	3.30	3.14	2.93
	Predictor Marginality - Köppen|GEnS									
bio02	−0.43	−0.39	−0.69	−0.65					−0.75	−0.72
bio03					0.44	0.39	0.77	0.73		
bio04					−0.47	−0.42	–	0.79		
bio06	–	0.36			0.48	0.45	0.75	0.73	0.81	0.79
bio07	−0.47	−0.42			−0.51	−0.45	−0.87	−0.85	−0.80	−0.77
bio11					0.41	0.38				
bio12									0.66	0.64
bio13	0.79	–								
bio14	0.42	0.36	0.61	0.55			0.83	–	0.72	0.67
bio17			0.55	0.50			0.76	0.75		
bio18			0.56	0.52						
bio21					−0.44	−0.40				
bio26					−0.38	−0.34				
bio28	0.43	0.37	0.56	0.51			0.73	0.72	0.70	0.66
bio29	0.43	0.36								
bio30			0.61	0.56			0.88	0.83		
bio32	0.42	0.34							0.66	0.61
bio33			0.61	0.56			0.86	0.80	0.66	0.62
bio34			0.62	0.57						
bio35	0.44	0.37			0.40	0.33				

The ENFA derived parameters are determined separately for each of the species boundary hypotheses and for all four species combined. The scores calculated across the Köppen-Geiger background are on the left and the GEnS scores on the right. The total marginality score will increase above 1 when considering all predictor variable marginality scores. Bio02 =  Mean diurnal temperature range (mean(period max-min)) (°C); Bio03 =  Isothermality (Bio02 ÷ Bio07); Bio04 =  Temperature seasonality (C of V); Bio06 =  Min temperature of coldest week (°C); Bio07 =  Temperature annual range (Max temperature of warmest week - Bio06) (°C); Bio11 =  Mean temperature of coldest quarter (°C); Bio12 =  Annual precipitation (mm); Bio14 =  Precipitation of driest week (mm); Bio17 =  Precipitation of driest quarter (mm); Bio18 =  Precipitation of warmest quarter (mm); Bio21 =  Highest weekly radiation (W m^−2^); Bio26 =  Radiation of warmest quarter (W m^−2^); Bio27 =  Radiation of coldest quarter (W m^−2^); Bio28 =  Annual mean moisture index; Bio29 =  Highest weekly moisture index; Bio30 =  Lowest weekly moisture index; Bio32 =  Mean moisture index of wettest quarter; Bio33 =  Mean moisture index of driest quarter; Bio34 =  Mean moisture index of warmest quarter; Bio35 =  Mean moisture index of coldest quarter.

Overlap in G-space was investigated using reciprocal distribution models (RDM; [13] Fitzpatrick *et al*., 2007), which are reciprocally projected ENMs calibrated on separate distribution datasets and geographic backgrounds [13]–[15]. Such models are then reciprocally projected between native and invasive or novel ranges to measure how well models transfer and describe both distributions. Ecological niche models were constructed with Maxent (version 3.3.2i; [58], [59]), a presence-background ENM method. Using Maxent (and other ENM methods) to predict the potential niche of novel environments requires model extrapolation, thus appropriate caution should be taken to limit potential problems that result from violating underlying assumptions on training data [23], [60], Maxent has been used widely for investigating distributions of different invasive and pest invertebrates and plants (e.g. [14]–[16], [26]) and was also applied to *B. invadens* [38]. For each predictor set we sampled 10 000 random points across each background, so that either every cell was accounted for, or we had good representation for each. We only examined the two datasets that were used in the PCAs; the two *B. dorsalis* models were projected to the background of *B. invadens* and *vice versa*. We then combined *B. dorsalis* with *B. papayae* and *B. philippinensis* to test against *B. invadens.* To test RDM performance, we used the reciprocal species occurrences as a test dataset and examined AUC_TEST_ (area under the receiver operating characteristic curve for test dataset) score. Typically, models with AUC values over 0.7 are performing well, with over 0.9 being excellent. Below 0.5 is considered no better than random (e.g [14]–[15]). For our RDMs, default Maxent parameters were used except that only hinge features were enabled (hinge features allow for a change in the gradient of the response, provide a “smoother” model when used alone (Maxent option), and are recommended for modelling invasive species; see [23], [61]) and models only constructed using the ‘wider’ Köppen-Geiger backgrounds. As an additional evaluation of model performance, we used the True Skill Statistic (TSS) [62] which ranges from −1 to +1, with values of +1 being perfect and ≤0 considered no better than random [62]. The TSS is threshold-dependent and was calculated using omission and commission rates set at a threshold of maximum sensitivity plus specificity. Like AUC, TSS weights sensitivity and specificity equally [62], this needs to be considered when evaluating false negative predictions (omission errors), and consequences of, for invasive species [18]. We aimed to reduce false negative predictions prior, by exploring model features in an attempt to smooth responses and increase transferability, and then use equal weights for evaluation.

### Range Expansion

To focus on the current range expansion into southern Africa we built ENMs (agains using Maxent) with a combination of background (2), predictor set (2) and species datasets (4: the species boundary hypotheses), We sought to reduce ENM complexity through ‘smoothing’ predictor responses in an attempt to increase transferability and avoid possible underprediction [26]. We also only enabled hinge features and set the regularization parameter (*β*) at 1, 2 and 5 to examine how increases in *β* affected model fit and prediction. Regularization is a process of smoothing the model fit through making it more regular in an attempt to avoid fitting a too complex model [61]. All other settings were left at default and we employed 10 000 background points. Final models were run with 10 cross-validation replicates and the AUC_TEST_ score examined. While AUC_TEST_ was appropriate for the RDMs (ENMs using independent test datasets, not split-dataset approach), the use of AUC may be problematic as an evaluation of ENMs attempting to describe the potential distribution (e.g. [63], [64]). So, to further evaluate model performance and rank complexity for each of the different ‘species’ datasets, we calculated sample size-corrected Akaike information criteria (AIC_c_) (using ENMTools; [65], [66]) to determine the lowest AICc value (coupled with a high AUC_TEST_ value). We considered all combinations of background choice, predictor set (for ENFA – with and without correlated pairs identified and removed) and the different *β* values for all models. We performed paired *t*-tests across AICc scores between each model constructed on each species dataset. As a final check we examined correlation between variable pairs using Pearson’s correlation coeffecient (*r)* for the chosen models across respective backgrounds and examined model performance when removing variables for any pair where *r* ≥0.75. Whilst Pearson’s *r* is only one measure of correlation between variables, it allowed for examination of linear correlations across the entire background area of our final predictor sets that may hamper model transferability.

To examine range expansion we projected the best performing ENM (selected through AICc approach) for each species boundary hypothesis to southern Africa and included a reconstructed De Meyer *et al.* [38] Maxent model. We evaluated the performance of these final models using TSS as before and measured niche breadth (B = Levin’s measure of niche breadth (inverse concentration): see [67]), and niche overlap (Schoener’s *D*) using ENMtools, for each of the ENMs below 14.78°S on the African mainland (the most southern locality from the De Meyer *et al.* [38] dataset) across the logistic output grids from Maxent. We also acquired positive trap identifications from an area that has displayed recent incursion of *B. invadens* in South Africa. This translated into 11 trap points, but these only represented four grid cells at the resolution of our predictor layers. To test how each of the four ENMs predicted the recent invasion of *B. invadens* into South Africa we examined the test AUC_TEST_ value using these trap data as an independent test dataset in Maxent.

## Results

### Bactrocera spp. Distributions

*Bactrocera invadens* and *B. dorsalis* s.s. are found across 10 different Köppen-Geiger climatic zones each, both occur in Asia, but *B. invadens* is also now widespread through Africa (Fig. 1a). Both *B. papayae* and *B. philippinensis* have restricted distributions in South East Asia, in tropical climate zones (Fig. 1a). For the GEnS background, *B. dorsalis* is found across 38 strata, *B. invadens* across 31 and *B. philippinensis* and *B. papaya* across 6 and 8, respectively. The climatic zones these strata fall into show that typically the species are found in hot or extremely hot climates with varying rainfall regimes, from moist through to arid (Fig. 1b).

### Background and Predictor Sets

The Köppen-Geiger defined backgrounds resulted in fewer climate zone classes and therefore wider geographic regions than did the backgrounds defined by occupied GEnS strata. The ENFA derived variables were different for each of the *Bactrocera* species (Table 1). There are no shared variables between the *B. dorsalis* and *B. invadens* datasets across both the Köppen-Geiger and GEnS backgrounds. *Bactrocera philippinensis* and *B. papayae* each share four variables with *B. dorsalis* ¸ and *B. papaya* shares four variables with *B. invadens*, while *B. philippinensis* only two (Table 1). *Bactrocera invadens* has the lowest scores for marginality and specialization (Table 1), indicative of a widespread species across a variety of habitats. *Bactrocera dorsalis* has also low marginality and specialization scores, though higher than *B. invadens*. *Bactrocera philippinensis* and *B. papayae* have high specialization scores (Table 1), reflective of the small distributions across only a few climatic zones (Figs. 1a, b). The specialization scores for the combined dataset of all species are the lowest, although the marginality score is still higher than for *B. invadens* alone (Table 1).

### Niche Overlap

**Figure 2:**
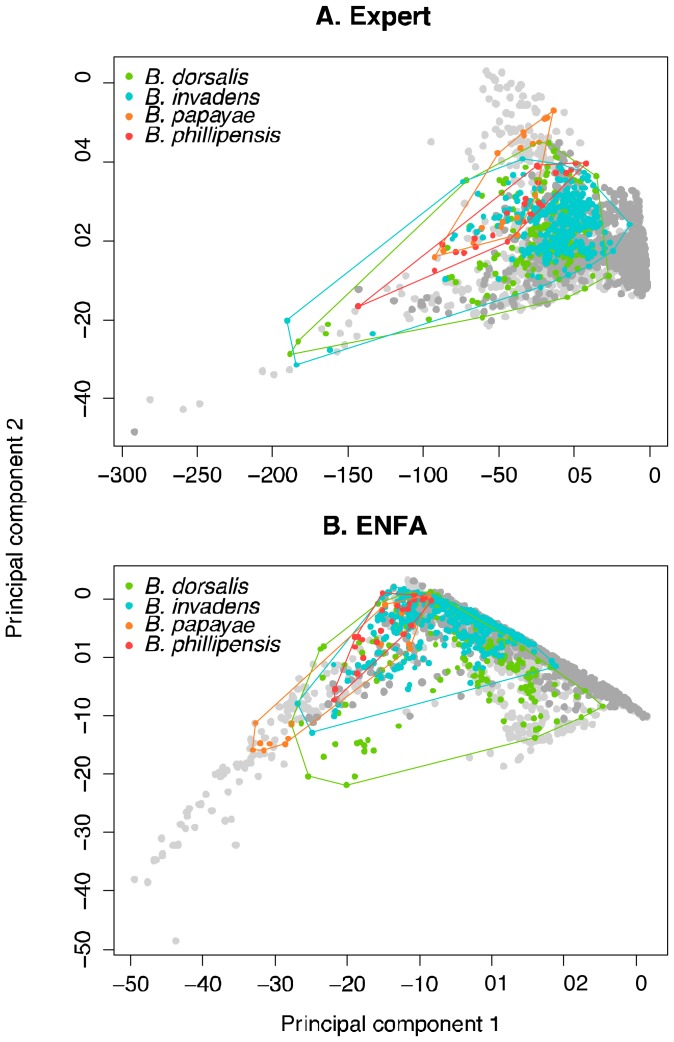
Principal components analysis (PCA) of four different *Bactrocera* spp. across different predictor variable sets. Light grey points represent 1000 random background points across the range of *B. dorsalis* s.s. and dark grey, *B. invadens*. **a)** PCA for “expert” predictor set. Proportion of variance for PC1 = 88.8% and for PC2 = 6.4%. **b)** PCA for ENFA driven predictor set (note: eight variables were loaded, most informative across the 4 “species”) Proportion of variance for PC1 = 68.4% and for PC2 = 24.6%.

When examined in E-space, the PCAs for both the expert predictor set (Fig. 2a) and the ENFA derived predictor set (Fig. 2b) display high overlap across the four species. The accessible E-space (represented as light and dark grey dots in Figs. 2a and b) across the *B. dorsalis* and *B. invadens* backgrounds form two largely overlapping clouds when plotted on the first two principal component axes, though displays clear divergence along the two axes, particularly for the light grey points depicting the background in Asia.

**Figure 3:**
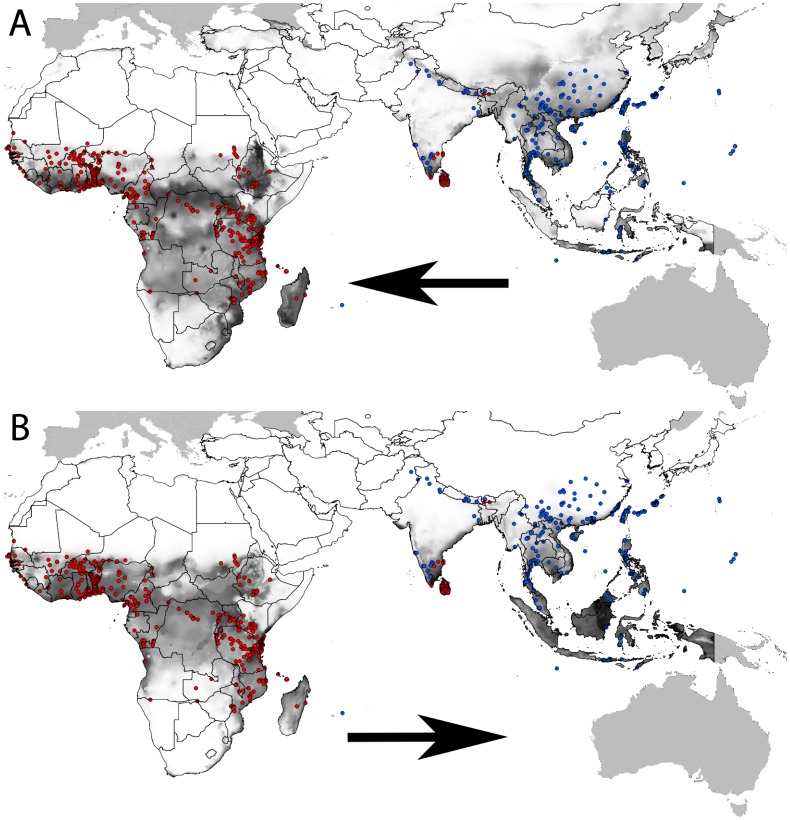
Reciprocal distribution models RDM for *B. dorsalis* s.s. + *B. philippinensis + B. papayae* (blue dots) and *B. invadens* (red dots). Ecological Niche Models shown here were constructed on ENFA-derived predictor sets as they had higher AUC_TEST_ and *D* scores than did those built on expert-driven predictor sets (see Table 2). Shading indicates suitability and solid grey areas are those that fall outside Asia and Africa. a) RDM trained on *B. dorsalis* + *B. papayae* + *B. philippinensis* distribution projected to the background of *B. invadens*, Model H (Table 2). b) RDM trained on *B. invadens* distribution projected to the background of *B. dorsalis* s.s. + *B. philippinensis* + *B. papayae*, Model E (Table 2).

As well as being high overlap in E-space there is also high overlap in G-space, as demonstrated through RDM transferability (Figs. 3a, b) and supported in high AUC_TEST_, TSS and niche overlap scores (Table 2). The RDMs for *B. invadens* and *B. dorsalis* show that each species is able to project across to the distribution of the other, but in particular *B. dorsalis* s.s. over to *B. invadens* (ENFA: AUC_TEST_ *= *0.84, *D* = 0.86; Expert: AUC_TEST_ = 0.80, *D* = 0.81) and this overlap even further enhanced by incorporating the points for *B. philippinensis* and *B. papayae* and projecting to Africa (ENFA: AUC_TEST_ *= *0.83, *D* = 0.91; Expert: AUC_TEST_ *= *0.845, *D* = 0.93). Combining the *Bactrocera dorsalis* species in Asia gives better prediction of the *B. invadens* range in Africa and gives high spatial congruence with this distribution. Schoener’s *D* values for *B. invadens* projected to the Asian background range from 0.51–0.68, indicating moderate success in projecting to this range, though not predicting the northern extent of *B. dorsalis* in Asia (Figs. 3b).

**Table 2:** RDM performance.

Model	Calibration	Project	Dataset	AUC_TRAIN_	AUC_TEST_	TSS	*D*
A	*B. invadens*	*B. dorsalis*	ENFA	0.888	0.762	0.559	0.51
B	*B. invadens*	*B. dorsalis*	Expert	0.881	0.683	0.480	0.51
C	*B. dorsalis*	*B. invadens*	ENFA	0.891	0.841	0.579	0.86
D	*B. dorsalis*	*B. invadens*	Expert	0.894	0.804	0.461	0.81
E	*B. invadens*	DPP	ENFA	0.888	0.787	0.568	0.76
F	*B. invadens*	DPP	Expert	0.881	0.731	0.494	0.68
G	DPP	*B. invadens*	ENFA	0.884	0.83	0.553	0.91
H	DPP	*B. invadens*	Expert	0.886	0.845	0.563	0.93

Models were assessed on their ability to predict the distribution of the other species with the AUC_TEST_ (area under the receiver operating characteristic curve) score (independent dataset not included in model construction). DPP = *B. dorsalis* + *B. philippinensis* + *B. papayae*. True Skill Statistic (TSS) values were calculated to evaluate model performance at the threshold of maximum training sensitivity plus specificity. Schoener’s *D* values are the overlap of the given model in the *projected* range where the reciprocal model was calibrated.

### Range Expansion

Our final models for the four datasets were selected on significantly lowest AICc (coupled with a high AUC_TEST_ score) (Table 3). Generally, the Köppen-Geiger background with the expert-driven predictor set yielded ENMs with higher performance, only separated on regularization changes (*B. dorsalis β = *2, *p*<0.05; All *β* = 5, *p*<0.05; *B. dorsalis* + *B. papaya* + *B. philippinensis β* = 2, *p*<0.005), except for *B. invadens* where the ENFA variables gave the lowest AICc value (*β* = 2, *p*<0.001) (Table 3). However, variables describing minimum temperature of the coldest month and annual temperature range are highly correlated therefore causing some spurious spatial predictions for this ENM, so we removed the latter variable *post hoc.* Generally, by increasing *β* to values of 2 or 5, the AICc values were also significantly lowered – further reducing model complexity (beyond selecting only hinge-features) increased model performance (Table 3). Coupled with significantly different AICc scores for all model selections (*p*<0.05) the mean AUC_TEST_ was >0.80, indicating high predictive ability given model conditions (Table 3). In addition our final models (bold in Table 3) all performed well with TSS values of: *B. invadens* = 0.602, *B. dorsalis* = 0.596, *B. dorsalis + B. philippinensis + B. papayae* = 0.607, All species = 0.532.

**Table 3:** Ecological Niche Model (ENM) performance for different *Bactrocera dorsalis* complex datasets.

		AICc				AUC_TEST_			
		Köppen-Geiger		GEnS		Köppen-Geiger		GEnS	
Species	*β*	Expert	ENFA	Expert	ENFA	Expert	ENFA	Expert	ENFA
***B. invadens***	1	10082.55	10115.73	10067.98	10161.67	0.870	0.879	0.848	0.857
	2	10015.31	**9948.31**	10040.87	10001.62	0.863	0.872	0.838	0.85
	5	10033.44	10024.97	10054.66	10084.71	0.855	0.861	0.827	0.837
***B. dorsalis***	1	5185.53	5778.47	5055.31	5691.30	0.869	0.846	0.877	0.852
	2	4847.52	5721.84	4869.70	5640.72	0.872	0.837	0.873	0.845
	5	**4785.07**	5370.73	4799.10	5329.23	0.868	0.818	0.873	0.828
**DPP**	1	6330.44	6564.84	6233.28	6628.09	0.867	0.907	0.867	0.892
	2	5975.67	6595.53	6008.80	6495.63	0.863	0.900	0.864	0.884
	5	**5942.12**	6569.79	5977.70	6530.93	0.858	0.892	0.860	0.874
**All**	1	16698.46	16095.50	16239.09	16514.54	0.841	0.851	0.816	0.826
	2	16044.53	16136.19	16185.38	16497.96	0.836	0.843	0.810	0.819
	5	**16016.01**	16170.28	16131.59	16471.56	0.831	0.830	0.805	0.804

DPP = *B. dorsalis* + *B. philippinensis* + *B. papayae*, All = *B. dorsalis* + *B. invadens* + *B. papaya* + *B. philippinensis* AICc = sample size corrected Akaike information criteria across 10 replicates, bold values represent significantly lowest AICc score (*p*<0.05); AUC_TEST_ = area under the receiver operating characteristic curve; mean across 10 cross-validated replicates. *β* = regularization parameter.

**Figure 4:**
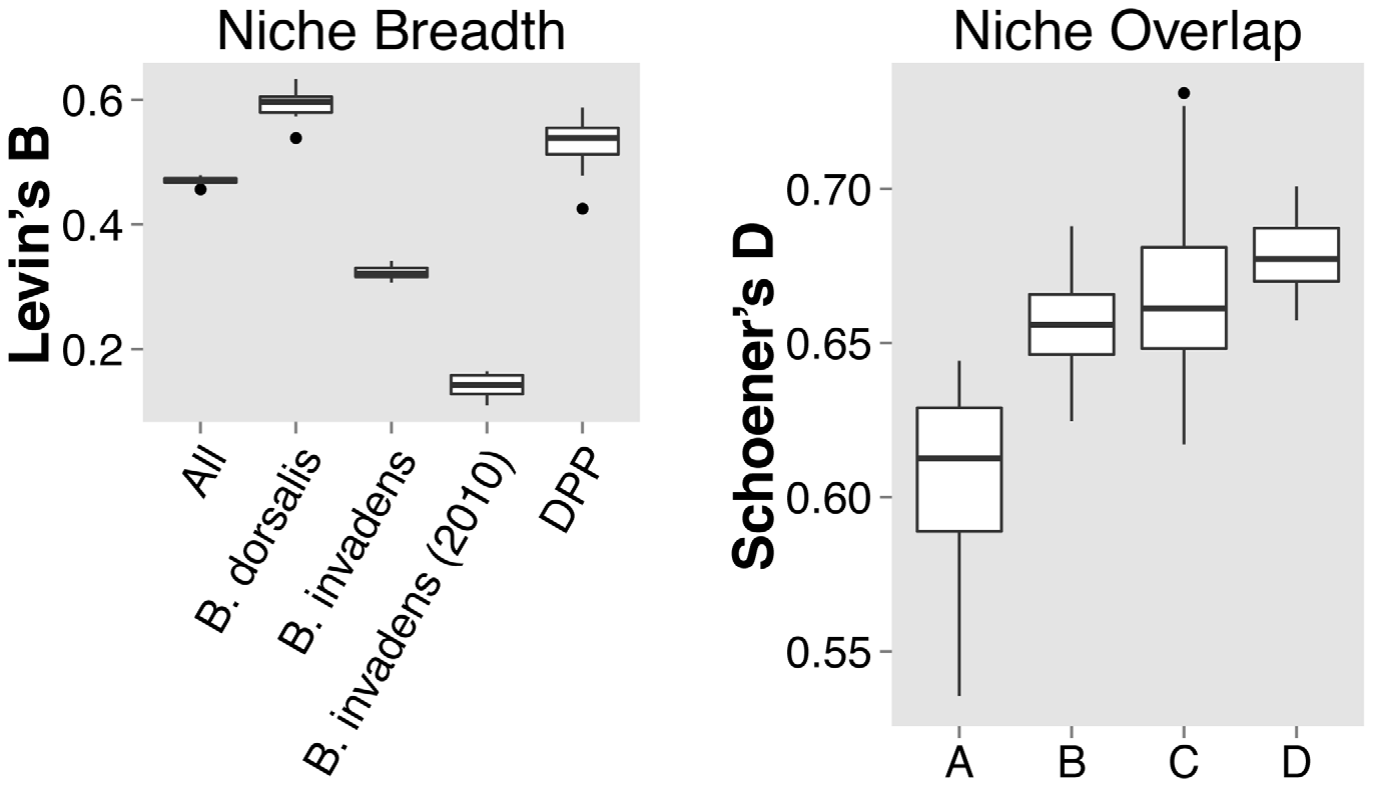
Niche metrics calculated for *Bactrocera* spp. Ecological Niche Models. **a)** Niche breadth (Levin’s B) for 10 replicates of each final ENM projected to mainland southern Africa (see Fig. 5a). Note: DPP = *B. dorsalis* + *B. philippinensis* + *B. papayae.* **b)** Niche overlap (Schoener’s *D*) between ENMs projected to mainland southern Africa (see Fig. 5a): A = *B. invadens* & *B. invadens* De Meyer *et al.* (2010); B = *B. invadens* & *B. dorsalis*; C = *B. invadens* & *B. dorsalis* + *B. philippinensis* + *B. papayae* (DPP); D = *B. invadens* & All four species combined.

Niche breadth in southern Africa was significantly highest for the *B. dorsalis* s.s. ENM (B = 0.59, *p*<0.001) (Fig. 4a). Niche breadth for the combined dataset (B = 0.47) and the dataset of *B. dorsalis + B. philippinensis + B. papayae* (B = 0.53) were significantly higher than for *B. invadens* (B = 0.36, *p*<0.001 in both cases). Our *B. invadens* ENM also had higher niche breadth than the De Meyer *et al*. [38] *B. invadens* ENM in southern Africa (*p*<0.001) (Fig. 4a). Pairwise comparisons of niche overlap in southern Africa between the final *B invadens* ENM and three other models revealed that the highest overlap was with the model considering all four species simultaneously (Comparison D, *D* = 0.68, *p*<0.001, Fig. 4b). Niche overlap between *B. invadens* and *B. dorsalis* was also high (Comparison B, *D* = 0.66, *p*<0.01) and consistent with niche breadth, there was higher overlap between *B. invadens* and the model with the other three species combined (Comparison C, *D* = 0.67, *p*<0.01). The De Meyer *et al.* [38] ENM and our *B. invadens* ENM had the lowest overlap (Comparison A, *D = *0.61, *p*<0.001, Fig. 4b).

**Figure 5:**
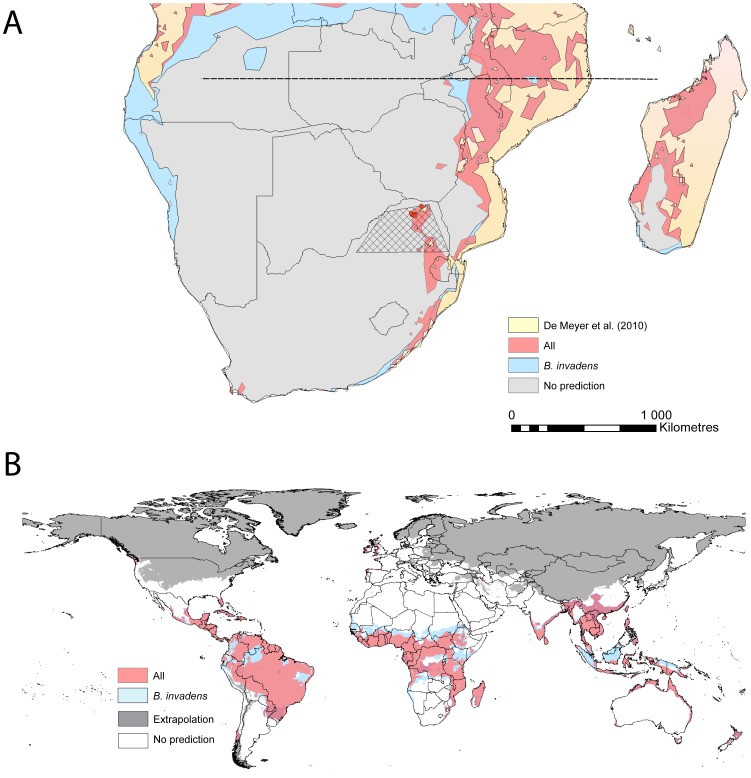
Final *Bactrocera* spp. Ecological Niche Models (ENMs) projected spatially a) Final ENMs projected to southern Africa to predict the range expansion of *B. invadens*. Hatched area = area affected by recent *B. invadens* incursions. Red points are known localities of trapped flies. Models displayed at a binary presence/absence threshold set at maximum training sensitivity plus specificity. **b)** Final ENMs projected to show global invasion potential of *Bactrocera invadens* and when considered as a single species with *B. dorsalis*, *B. philippinensis* and *B. papayae*. Shading indicates variables outside training range and extrapolation (calculated with the multivariate environmental similarity surface (MESS) analysis in Maxent [26]). Models displayed at a binary presence/absence threshold set at maximum training sensitivity plus specificity.

Overall, the final models for the *B. invadens* dataset and the all species combined dataset predict slightly different geographic area in Africa, particularly in the northern parts of the African range for *B. invadens* and in the southern parts of the range for the combined dataset (Fig. 5a). The De Meyer *et al.* [38] model predicts a more conservative distribution than these two models (Fig. 5a). The 11 points (4 grid cells) from the recent invasion of *B. invadens* in South Africa, all fall within a small area in the Limpopo province (hatched area, Fig. 5a). Consistent with the results for niche breadth, the AUC_TEST_ values for these points were low for *B. invadens* (AUC_TEST_ = 0.547), but then high for all species combined (AUC_TEST_ = 0.844) and very high for *B. dorsalis* (AUC_TEST_ = 0.937) and *B. dorsalis* + *B. philippinensis* + *B. papayae* (AUC_TEST_ = 0.924) ENMs. While these AUC values should be interpreted cautiously given the low number of test points they do provide an indication of ENM performance for predicting this recent range expansion. The predicted global invasion potential of *B. invadens* and all four species combined is shown in Figure 5b.

## Discussion

The recent range expansion and invasion of *Bactrocera invadens* into South Africa is a major concern for fruit growing industries within the country. Through ENMs and niche-exploration methods, we elucidated species-environment relationships and likely drivers of the geographical expansion of *B. invadens.* In answer to the questions posed by our study aims, *B. invadens* displays a highly overlapping niche in terms of both E-space and G-space with *B. dorsalis* s.s. (and *B. philippinensis* and *B. papayae*), supporting evidence that these species may indeed be conspecific. Secondly, the range expansion and invasion of *Bactrocera invadens* into South Africa is better explained through incorporating the species-environment relationships of these other members of the *B. dorsalis* complex. Thirdly, these results provide important information to predict the ongoing invasion of these *Bactrocera dorsalis* complex members and help direct recommendations for global management of these high risk species.

High overlap in both E- and G-space, and for both predictor sets used, is consistent with the hypothesis for *Bactrocera invadens*, *B. philippinensis* and *B. papayae* to be conspecific with *B. dorsalis* s.s. It is evident however, that E-space changes between ranges, as climatic variables are often anisotropic across large geographic extents like the backgrounds employed here [68]. This was largely visible through our PCA biplots, and may help explain the low transferability of the *B. invadens* RDM to Asia, rather than a niche shift as concluded elsewhere (e.g. [13]–[15]). The incomplete transferability may also be due to *B. invadens* being in a state of range expansion: that *B. dorsalis* s.s. is found in more strata from the GEnS analysis may be further indicative of this suggestion. The advantages of updating distribution data is demonstrated by geographic differences and lowest niche overlap between the De Meyer *et al*. [38] model and the ENMs explored herein. Information from trap catches (there are now over 3000 Methyl Eugenol traps throughout South Africa [69]), including seasonality and abundance, should provide essential data to construct dispersal models, revisit ENMs, and further understand the rate at which *B. invadens* is spreading.

Without having true absence data to calibrate our ENMs, we are providing an assessment of invasion potential rather than the actual distributions for *B. invadens*/*dorsalis* [16]. By incorporating information from other members of the *B. dorsalis* complex into the *B. invadens* ENMs, some insight into the recent range expansion into South Africa can be achieved. Importantly, rather than a niche shift for *B. invadens*, range expansion is likely to be a single conspecific invader filling its potential niche. The differences in overlap and geographic extent between the *B. invadens* and the combined models may be due to sub-taxon consideration of datasets [23], [30]. The *B. invadens* model and the combined model of the four species may reflect differences in ecology and thus provide complementary information for determining invasion potential [22]. To describe invasion potential we also attempted to increase transferability and minimize false negative predictions through reducing model complexity (e.g. feature selection). Associated error is thus more likely to fall on the side of over-prediction (commission error) rather than under-prediction (omission error) and this is likely to be a more desirable outcome when predicting the spread of a rapidly expanding species, though caution is required when translating this to management practices [18].

Invasive species that occupy large geographic extents may be modelled effectively through generalised bioclimatic backgrounds, as we found that the Köppen-Geiger was less restrictive than the GEnS background, resulting in higher model performance (or presence/background discrepancy). While use of wide backgrounds has typically been found to show lower transferability [70], model performance is affected by either too wide or too narrow a background [47]. A background based on dispersal would likely provide a useful test against these bioclimatic backgrounds, but quantifying and accurately modelling both active *and* passive dispersal remains challenging at present, due partly to the dispersal of tephritids through factors such as human-assisted dispersal [71]. It is likely that the GEnS selected backgrounds are suited to ENM applications for niches that are not under rapid change, such as conservation and biogeography monitoring-type analyses [52]. The fact that ENMs that were constructed on the expert-driven predictor variable set generally performed better than our ENFA one(s) provides good support for variable selection to be based on knowledge of physiological (or other functional) limits that define distributions [72]. However, often such knowledge is not present for an invasive species, and as our *Bactrocera* ENMs built on ENFA selected variables gave high performance, transferability and spatial congruence with the expert-driven predictor sets, we recommend that ENFA provides a valid alternative where such functional information is lacking, given that correlated predictors are identified.

Ecological niche models are useful tools for understanding invasion potential on condition that the weaknesses are identified and future research plans are centred on testing processes outside model capabilities (e.g. biotic interactions, dispersal and adaptation) (see [10]). For instance, competition has been observed between *Bactrocera invadens* and the indigenous *Ceratitis cosyra*, although in this case *B. invadens* was able to outcompete *C. cosyra* [73]. Likewise, thermal tolerance traits have been shown to differ between closely related tephritids, *Ceratitis capitata* and *C. rosa*, and this may translate into a competitive advantage to the former or a broader thermal niche [74]. By using established thermal tolerance and desiccation protocols it should be possible to test whether overlaps in E-space are related to a high degree of physiological similarity between the species [74], or if there are any shifts in physiological traits [27]. Testing for local adaptation in functional traits (such as physiological tolerances or host plant switching) can also reveal evolutionary processes that facilitate range expansion (e.g. [28]). Phenological studies and abundance data could be used to predict outbreaks and persistence of *B*. *invadens* across the geographic area of invasion potential (e.g. *Ceratitis rosa* [75]). For example, such information could be used to revise the existing *Bactrocera dorsalis* CLIMEX model [37] to examine invasion processes of *B. invadens*.

From previous climate-based models [37], [38], both *Bactrocera invadens* and *B. dorsalis* s.s. appeared to have the potential to independently invade large geographic areas and, given the global invasion of other tephritids to date (e.g. *C. capitata* [53]), this seems like a reasonable assertion. Our results support a growing body of evidence that species boundaries in the *B. dorsalis* complex may require revision (e.g. [32], [33], [35], [36]). Thus, we suggest that considering these *B. dorsalis* complex members separately has led to the underprediction of the invasive potential in both South Africa and globally. Proper management of pest invertebrates relies on correct identification of species, and due to the economic importance of these species, quarantine and management recommendations for *B. invadens* and *B.dorsalis* s.s. may need to be revised [32], [36]. However, we agree with Shutze *et al.* [33], [36] that behavioural and cytogenetic studies need to be completed before complete taxonomic revision.
